# *Sphingobacterium hotanense* Infections in Immunocompromised Patients, United States

**DOI:** 10.3201/eid3201.251290

**Published:** 2026-01

**Authors:** Khalid Abu-Zeinah, Benjamin D. Lueck, Shane A. Fuentes, Emily Puumala, Omar M. Abu Saleh

**Affiliations:** Mayo Clinic, Rochester, Minnesota, USA

**Keywords:** Sphingobacterium hotanense, bacteria, bacteremia, cellulitis, Sphingobacterium, antibacterial agents, fluoroquinolones, Minnesota, United States

## Abstract

*Sphingobacterium hotanense* is a gram-negative bacillus identified in 2013 from soil samples that rarely causes infection in humans. We describe 2 cases of *S. hotanense* bacteremia secondary to skin and soft tissue infection in immunocompromised patients in Minnesota, USA, highlighting *S. hotanense* as a potential pathogen in immunocompromised hosts with environmental exposure.

*Sphingobacterium hotanense* is a strictly aerobic, gram-negative bacillus first isolated in 2013 from soil in China (*1*). The *Sphingobacterium* genus includes >50 species typically found in soil, compost, and aquatic habitats (*2*,*3*). Documented infections with *Sphingobacterium* spp. in humans are rare (*2*,*3*), manifesting mostly as skin and soft tissue infections (SSTI) in immunocompromised patients (*3*); we found limited reports of *S. hotanense* infections in the literature (*2*). We describe 2 cases of *S. hotanense* bacteremia secondary to SSTIs in Minnesota, USA.

The Mayo Clinic Institutional Review Board (IRB) acknowledged that based on the responses submitted for our activity through the Mayo Clinic Human Subjects Research Wizard tool, and in accordance with the Code of Federal Regulations, 45 CFR 46.102, this study did not require IRB review.

## The Study

**Figure 1:**
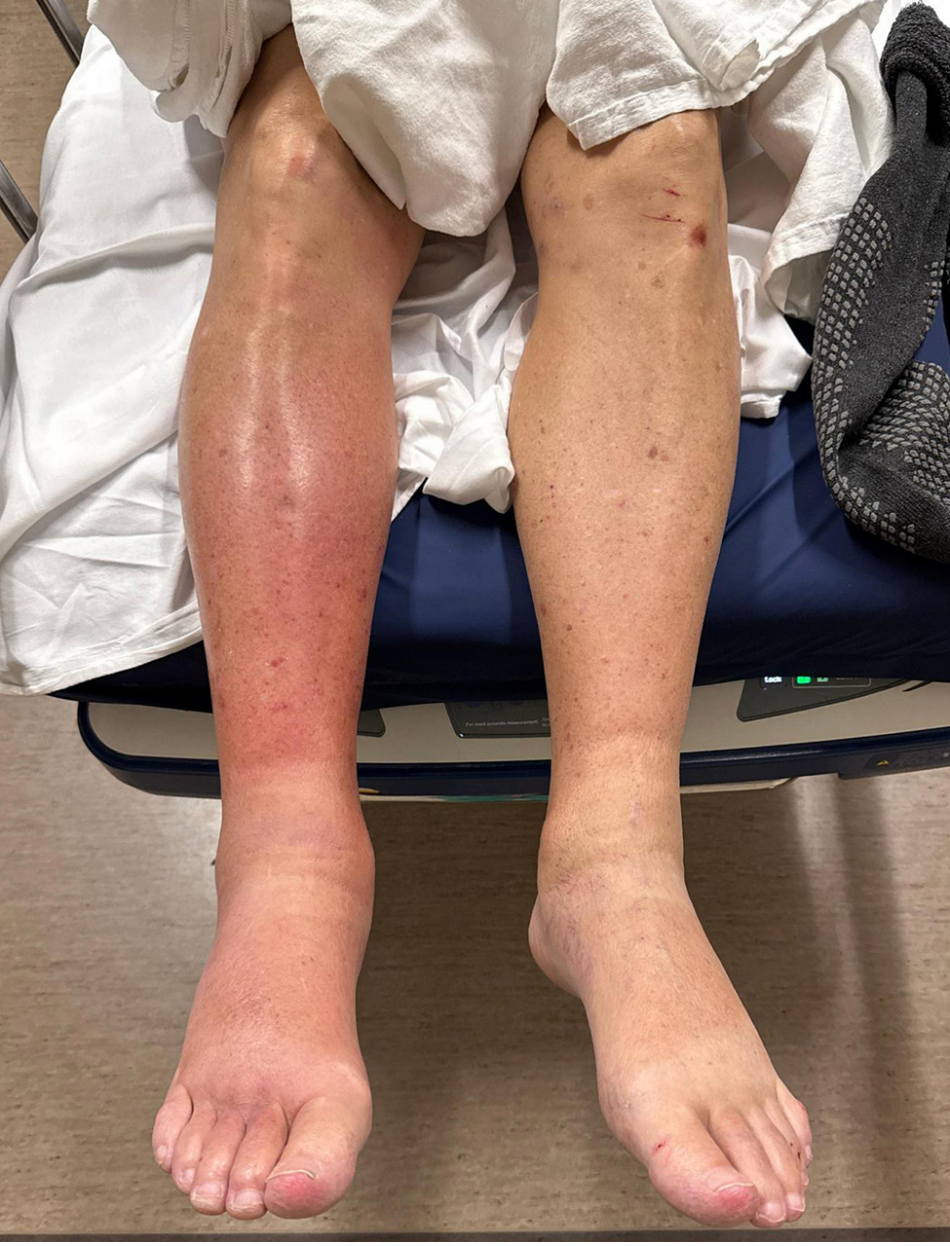
Lower extremities of patient in case 1 in report of *Sphingobacterium hotanense* infections in immunocompromised patients with skin and soft tissue infections, Minnesota, USA. Erythema and swelling of the right lower leg and foot are shown. No overt signs of abscess, necrosis, or purulence were noted at the time of examination.

Case 1 was in a 78-year-old man who sought care for acute right lower extremity pain. His medical history included ulcerative colitis treated with mesalamine, cirrhosis from primary sclerosing cholangitis, and iron deficiency anemia from bleeding portal gastropathy. Five days earlier, the patient went fishing in Montana, USA, where he walked barefoot on riverbeds and fell in soil. No other travel history or animal exposure was reported. The patient was febrile (38.6°C); examination revealed erythematous right lower extremity (Figure 1) and abdominal tenderness. HIV test was negative. Computed tomography (CT) imaging of the abdomen demonstrated moderate ascites. Medical staff administered intravenous ceftriaxone to treat cellulitis and possible spontaneous bacterial peritonitis (SBP); however, subsequent diagnostic paracentesis was not suggestive of SBP.

**Figure 2:**
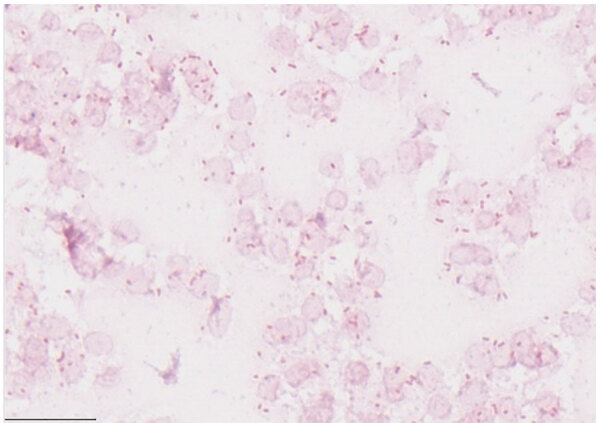
Gram stain of positive blood culture from patient in case 1 in report of *Sphingobacterium hotanense* infections in immunocompromised patients, Minnesota, USA. Numerous gram-negative bacilli are shown (pink rods), representing *S. hotanense* infection. Scale bar indicates 20 μm.

Blood cultures grew gram-negative rods within 12 hours, and antimicrobial drugs were switched to intravenous piperacillin/tazobactam. After 18 hours, speciation identified *S. hotanense* (Figure 2) in 2 of 3 aerobic culture bottles of 2 sets, with antimicrobial susceptibilities (Table). Bacteremia was attributed to SSTI. The patient remained on intravenous piperacillin/tazobactam for 72 hours and then was discharged on a 7-day course of oral levofloxacin; outpatient follow-up visit showed resolution of cellulitis.

**Table:** Antimicrobial susceptibility profile of isolates from blood cultures of 2 patients in report of *Sphingobacterium hotanense* infection in immunocompromised patients, Minnesota, USA

Antimicrobial drug	MIC, µg/mL	Susceptibility
Amikacin	>32	Resistant
Aztreonam	16	Intermediate
Cefepime	<2	Susceptible
Ceftazidime	<4	Susceptible
Ciprofloxacin	<0.25	Susceptible
Gentamicin	>8	Resistant
Levofloxacin	<0.5	Susceptible
Meropenem	<0.12	Susceptible
Piperacillin/tazobactam	<8/4	Susceptible
Tobramycin	>8	Resistant
Trimethoprim/sulfamethoxazole	<0.5/9.5	Susceptible

**Figure 3:**
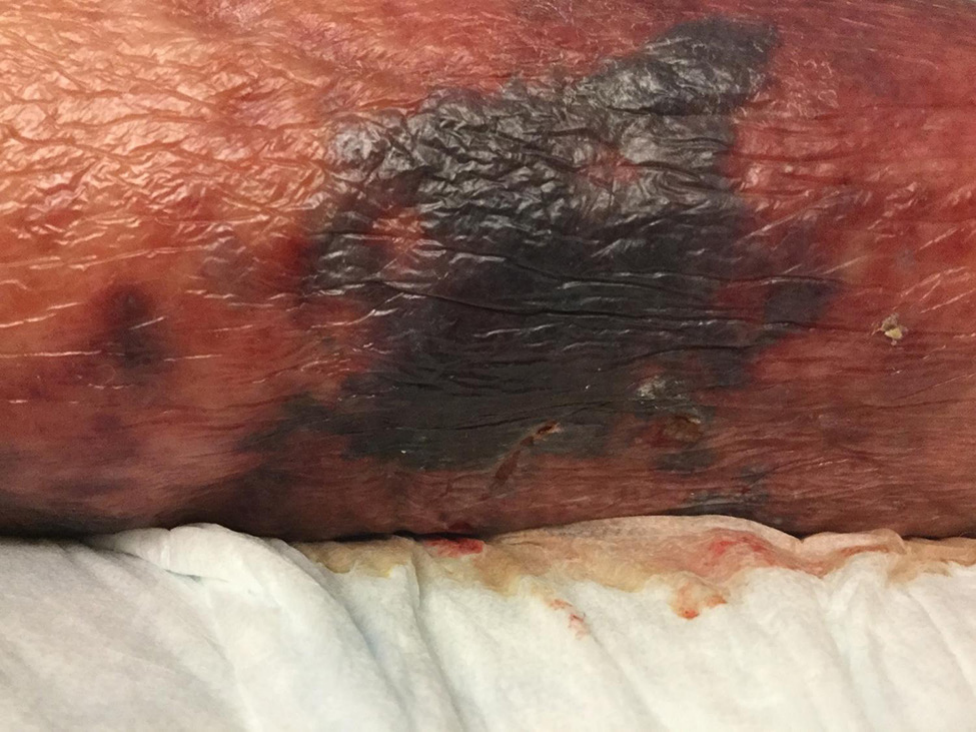
Right lower leg of patient in case 2 in report of *Sphingobacterium hotanense* infection in immunocompromised patients, Minnesota, USA*,* demonstrating extensive skin discoloration with surrounding erythema and increased drainage from wound (not visible).

Case 2 was in a 75-year-old man who sought care for acute right lower extremity pain; his lethargic state limited the ability to obtain a detailed medical history. His medical history included HIV infection managed with abacavir/dolutegravir/lamivudine; viral load was undetectable but CD4 count low (93/μL), attributed to concomitant myelodysplastic syndrome. He had a previous diagnosis of remitting seronegative systemic synovitis managed with chronic prednisone (5 mg/d), and had lower extremity stasis dermatitis with chronic open wounds. He was afebrile but hypotensive (91/60 mm Hg). Examination revealed erythematous right lower extremity (Figure 3). Laboratory studies revealed elevated lactate (5.6 mmol/L). CT imaging of the right leg demonstrated subcutaneous edema without abscesses or gas. He was admitted to the intensive care unit for vasopressor treatment and received intravenous vancomycin, ceftriaxone, and clindamycin. 

Blood cultures grew gram-negative bacilli after 12 hours, which was attributed to SSTI in his right leg. The antimicrobial regimen was narrowed to intravenous ceftriaxone and metronidazole. After 24 hours, *S. hotanense* was identified in 2 of 3 aerobic culture bottles from 1 set and in 1 bottle from a second set. Antimicrobial susceptibility was similar to that of case 1 (Table); intravenous ciprofloxacin was added to his regimen. Forty-eight hours after admission, his pain progressed, and operative debridement revealed extensive myonecrosis, necessitating above-knee amputation. Postoperative antimicrobial treatment was intravenous cefepime for 7 days. Intraoperative tissue cultures grew *Enterococcus faecalis* and *Enterobacter cloacae*; no additional antimicrobials were initiated because infection source was adequately controlled. Repeat blood cultures after the 7-day course of treatment indicated resolution of bacteremia. However, the patient then experienced acute pancreatitis, pulmonary embolism, candidemia, and pulmonary aspergillosis and died.

Blood cultures tested positive by BD BACTEC (Becton Dickinson, https://www.bd.com); we identified *S. hotanense* in our laboratory after subculture on standard growth media at 35°C with 5%–7% CO_2_ (Appendix Figure). *Sphingobacterium* spp. are not detected by rapid syndromic assays, including commercial multiplex PCR panels; the optimal method of species-level identification is diagnostic mass spectrometry or sequencing of the variable regions of the 16S rRNA gene. We identified isolates from case 1 and case 2 using matrix-assisted laser desorption/ionization time-of-flight mass spectrometry (Bruker Daltonics, https://www.bruker.com) based on Biotyper numeric score of 2.00–3.00 with >10% score separation from the next best score. Given the rarity of *Sphingobacterium* organisms in human infections, data on performance of commercial automated identification systems remain limited. In smaller or resource-limited laboratories, phenotypic identification may be guided by the biochemical profile of *Sphingobacterium* spp. (*4*).

We performed drug susceptibility testing using the agar dilution method to determine the MIC of antimicrobial drugs. We used reporting guidelines and breakpoints for gram-negative bacilli that are non-Enterobacterales, nonfastidious, and nonfermentative according to Clinical and Laboratory Standards Institute guidelines (*5*) to determine *S. hotanense* susceptibility. On retrospective evaluation, we identified 10 *S. hotanense* isolates referred from external institutions during July 2020–July 2025 (Appendix).

*Sphingobacterium* spp. are rarely implicated in human infections, typically affecting elderly or immunocompromised hosts (*3*). Reported infections typically involve SSTI with or without bacteremia (*3*); however, acute cholangitis (*6*), respiratory tract infections (*7*), septic arthritis (*8*), and meningitis have also been reported (*9*).

One documented case report from Europe described *S. hotanense* bacteremia secondary to cellulitis after a rooster scratch (*2*). Here, we report 2 additional cases of *S. hotanense* bacteremia from SSTI in immunocompromised hosts, consistent with previous reports. Case 1 involved a cirrhotic patient who had likely cirrhosis-associated immune dysfunction (*10*); exposure to river water and soil likely served as the source of inoculation (*1*). Case 2 involved a patient who had multiple immunocompromising conditions, including HIV, myelodysplastic syndrome, and corticosteroid use; lower-extremity wounds likely served as the port of entry.

The patient in case 1 had uncomplicated recovery after antimicrobial drug treatment. The patient in case 2 experienced a more severe course requiring above-knee amputation for necrotizing SSTI. In case 2, intraoperative tissue cultures grew *Enterococcus faecalis* and *Enterobacter cloacae*, consistent with the usual polymicrobial nature of necrotizing SSTI (*11*). The absence of *S. hotanense* from tissue cultures may reflect preoperative antimicrobial exposure affecting culture yield, given that no other source of bacteremia was identified.

When *S. hotanense* was isolated in 2013, antimicrobial susceptibility testing revealed resistance to ampicillin and tetracycline but susceptibility to ceftazidime (*1*). In subsequent reports, the organism demonstrated susceptibility to β-lactams, fluoroquinolones, and trimethoprim/sulfamethoxazole and resistance to aminoglycosides (*2*). The susceptibility patterns of the *S. hotanense* isolates in the cases we describe were similar to those previously reported, including sensitivity to broad-spectrum β-lactams such as ceftazidime, cefepime, piperacillin/tazobactam, and carbapenems as well as to fluoroquinolones and trimethoprim/sulfamethoxazole. Given the rarity of *S. hotanense* infections, antimicrobial selection should be guided by in vitro susceptibility results; piperacillin/tazobactam or cefepime are reasonable empiric choices and fluoroquinolones appropriate oral step-down options. Additional considerations include underlying conditions, drug allergies, and concern for polymicrobial infection.

Identification of *S. hotanense* in both cases we report was possible because we obtained blood cultures. Blood culture yield in cellulitis is generally low (<5%); cultures are therefore reserved for patients with high-risk features, such as sepsis, necrotization, immunosuppression, immersion injuries, or animal bites (*12*). Because cellulitis is often treated empirically without obtaining blood cultures, it might be that *S. hotanense* SSTI is more common than currently recognized but underdiagnosed because of infrequent microbiologic testing.

## Conclusions

*S. hotanense* is a rare cause of SSTI. Given its environmental reservoir, clinicians should maintain suspicion for *S. hotanense* in immunocompromised patients experiencing SSTI, especially with recent environmental exposure. More frequent microbiologic testing in select high-risk cases could reveal this organism as an underrecognized cause of infection.

Appendix Additional information for report of *Sphingobacterium hotanense* infections in immunocompromised patients, Minnesota, USA.

## References

[1] Xiao T, He X, Cheng G, Kuang H, Ma X, Yusup K, et al. *Sphingobacterium hotanense* sp. nov., isolated from soil of a *Populus euphratica* forest, and emended descriptions of *Sphingobacterium daejeonense* and *Sphingobacterium shayense.* Int J Syst Evol Microbiol. 2013;63:815–20. 10.1099/ijs.0.030155-0 22611196

[2] Kroumova V, Rossati A, Bargiacchi O, Garavelli PL, Camaggi A, Caroppo S, et al. From soil to blood: first human case of *Sphingobacterium hotanense* bacteraemia. Infez Med. 2017;25:75–6. 28353460

[3] Nemoto D, Hitomi S, Moriyama Y, Iwamoto K, Saito K. Cellulitis complicated with bacteremia due to *Sphingobacterium* species: a report of two cases and a literature review. Intern Med. 2019;58:2573–6. 10.2169/internalmedicine.2178-18 31118372 PMC6761337

[4] Cools P, Nemec A. Abeele A-Mvd, Kämpfer P. *Acinetobacter, Chryseobacterium, Moraxella, Branhamella* and other nonfermentative gram-negative rods. In: Carroll KC, Pfaller MA, editors. Manual of clinical microbiology, 13th edition. Washington: John Wiley & Sons, Inc; 2023. p. 1–33.

[5] Clinical and Laboratory Standards Institute. Performance standards for antimicrobial susceptibility testing, 35th edition. Supplement M100. Wayne (PA): The Institute; 2025.

[6] Akazawa N, Itoh N, Morioka H, Ogata T, Ishibana Y, Murakami H, et al. Cholangitis with *Sphingobacterium multivorum* and *Acinetobacter junii* bacteremia in a patient with gastric cancer: A case report. J Infect Chemother. 2022;28:1419–23. 10.1016/j.jiac.2022.06.005 35718261

[7] Lambiase A, Rossano F, Del Pezzo M, Raia V, Sepe A, de Gregorio F, et al. *Sphingobacterium* respiratory tract infection in patients with cystic fibrosis. BMC Res Notes. 2009;2:262. 10.1186/1756-0500-2-262 20030840 PMC2805677

[8] Mendes MD, Cavallo RR, Carvalhães CH, Ferrarini MA. Septic arthritis by *Sphingobacterium multivorum* in imunocompromised pediatric patient. Rev Paul Pediatr. 2016;34:379–83. 10.1016/j.rpped.2015.12.001 26915918 PMC5178126

[9] Abro AH, Rahimi Shahmirzadi MR, Jasim LM, Badreddine S, Al Deesi Z. *Sphingobacterium multivorum* bacteremia and acute meningitis in an immunocompetent adult patient: a case report. Iran Red Crescent Med J. 2016;18:e38750. 10.5812/ircmj.38750 28144466 PMC5256040

[10] Rodríguez-Negrete EV, Gálvez-Martínez M, Sánchez-Reyes K, Fajardo-Felix CF, Pérez-Reséndiz KE, Madrigal-Santillán EO, et al. Liver cirrhosis: the immunocompromised state. J Clin Med. 2024;13:5582. 10.3390/jcm13185582 39337069 PMC11432654

[11] Dhanasekara CS, Marschke B, Morris E, Kahathuduwa CN, Dissanaike S. Global patterns of necrotizing soft tissue infections: A systematic review and meta-analysis. Surgery. 2021;170:1718–26. 10.1016/j.surg.2021.06.036 34362585

[12] Stevens DL, Bisno AL, Chambers HF, Dellinger EP, Goldstein EJ, Gorbach SL, et al.; Infectious Diseases Society of America. Practice guidelines for the diagnosis and management of skin and soft tissue infections: 2014 update by the infectious diseases society of America. Clin Infect Dis. 2014;59:147–59. 10.1093/cid/ciu444 24947530

