# Supplementary material for: Sphingobacterium hotanense Infections in Immunocompromised Patients, United States
Source: Emerg Infect Dis. 2026 Jan;32(1):118–21. doi: 10.3201/eid3201.251290 (PMC12870043; doi:10.3201/eid3201.251290)
Supplement: Appendix — Additional information for report of Sphingobacterium hotanense infections in immunocompromised patients, Minnesota, USA. [file 25-1290-Techapp-s1.pdf]

Article DOI: https://doi.org/10.3201/eid3201.251290

EID cannot ensure accessibility for supplementary materials supplied by authors. Readers who have difficulty accessing supplementary content should contact the authors for assistance.

Sphingobacterium hotanense Infections in Immunocompromised Patients, United States

Appendix

Lab Methods

When grown on sheep's blood agar and chocolate blood agar (Appendix Figure, panels A and C), *S. hotanense* colonies are cream to yellow in color, convex, and circular with entire margins. Isolates also grow on Gram-negative selective eosin methylene blue (EMB) agar (Appendix Figure, panel B), appearing pink to colorless, indicating their inability to ferment lactose.

Susceptibility Testing

Between July 2020 and July 2025, 10 *S. hotanense* isolates were identified and tested for antimicrobial susceptibility in the Division of Clinical Microbiology at our institution, which performs both clinical and reference laboratory functionality. These isolates were referred to our laboratory for identification from external, client institutions based across the United States and globally. As such, we do not have access to clinical information for the patients from which the isolates were cultured, apart from susceptibilities of the isolate. Of the susceptibilities reported, 100% were susceptible to the β-lactams tested (ceftazidime, cefepime, meropenem, and piperacillin-tazobactam), the fluoroquinolones tested (levofloxacin, ciprofloxacin), and trimethoprim-sulfamethoxazole. Consistent with the published literature, all isolates tested in this 5-year cohort were resistant to the aminoglycosides tested (amikacin, gentamicin, tobramycin) (*1*,*2*). We noted variable susceptibility to aztreonam, with 30% susceptible, 30% intermediate, and 40% resistant. In our laboratory, first- through third-generation cephalosporins were not included in the susceptibility panel. Although CLSI provides breakpoints for representatives of

all cephalosporin generations within the non-*Enterobacterales* group (to which *Sphingobacterium hotanense* belongs), our laboratory follows CLSI M100 Table 1 guidance, which outlines tiers of antimicrobial agents recommended for routine reporting. Accordingly, only tier 1 and 2 agents are included in our testing panel, which prioritizes agents with demonstrated clinical relevance, reliable interpretive criteria, and expected activity for the organism group (*3*,*4*).

References

1. Xiao T, He X, Cheng G, Kuang H, Ma X, Yusup K, et al. *Sphingobacterium hotanense* sp. nov., isolated from soil of a *Populus euphratica* forest, and emended descriptions of *Sphingobacterium daejeonense* and *Sphingobacterium shayense.* Int J Syst Evol Microbiol. 2013;63:815–20. PubMed https://doi.org/10.1099/ijs.0.030155-0

2. Kroumova V, Rossati A, Bargiacchi O, Garavelli PL, Camaggi A, Caroppo S, et al. From soil to blood: first human case of *Sphingobacterium hotanense* bacteraemia. Infez Med. 2017;25:75–6. PubMed

3. Higgins CS, Murtough SM, Williamson E, Hiom SJ, Payne DJ, Russell AD, et al. Resistance to antibiotics and biocides among non-fermenting Gram-negative bacteria. Clin Microbiol Infect. 2001;7:308–15. PubMed https://doi.org/10.1046/j.1198-743x.2001.00253.x

4. Clinical and Laboratory Standards Institute (CLSI). Performance standards for antimicrobial susceptibility testing. 35th ed. CLSI supplement M100. Wayne (PA): Clinical and Laboratory Standards Institute; 2025.

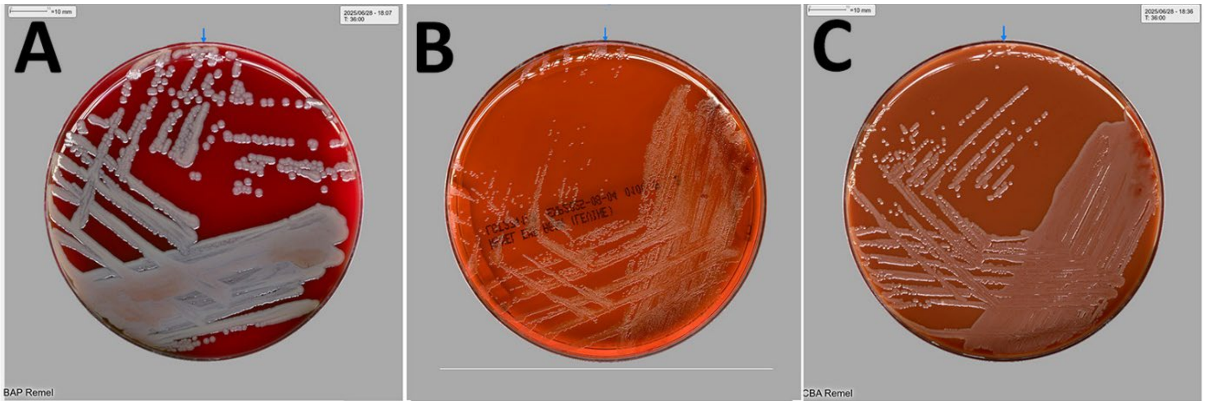

Appendix Figure. Growth of *Sphingobacterium hotanense* on various culture media after 36 hours of incubation. A) Blood agar showing smooth, creamy, opaque colonies with non-hemolytic growth. B) Eosin methylene blue (EMB) agar demonstrating minimal colony pigmentation (non-lactose fermenting). C) Chocolate agar with robust growth of moist, spreading colonies.
